# Drug and therapeutics committee interventions in managing irrational drug use and antimicrobial stewardship in China

**DOI:** 10.3389/fphar.2022.829408

**Published:** 2022-07-22

**Authors:** Jing Yang, Lei Zheng, Yu-Yao Guan, Yong-Tao Lv

**Affiliations:** ^1^ School of Medicine and Pharmacy, Ocean University of China, Qingdao, China; ^2^ Department of Pharmacy, Shandong Medical College, Jinan, China; ^3^ Department of Pharmacy, Shandong Provincial Third Hospital, Cheeloo College of Medicine, Shandong University, Jinan, China

**Keywords:** drug and therapeutics committees, rational drug use, management measures, drug cost control, clinical pharmaceutical care

## Abstract

**Aim:** This study aimed to investigate the key points in the transformation of the functions of the Drug and Therapeutics Committee (DTC) of the Shandong Provincial Third Hospital and how to provide full authority to its role in the control of rational drug use, especially in the management of antibiotic use.

**Method:** A prescription review management group, antimicrobial stewardship group, and rational drug use service group were established under the DTC. From January 2016 to December 2021, each group played a role in promoting rational drug use and antimicrobial stewardship. In addition, we performed statistics on typical management cases, irrational drug use, bacterial resistance rate, and drug costs from 2015 to 2021 to evaluate the effect of management by the DTC.

**Results:** Intervention by the DTC led to a significant reduction in prescribing errors (71.43%, *p* < 0.05), the intervention acceptance rate increased by 16.03%, and the problem solved rate increased by 32.41% (*p* < 0.05). Resistance rates of general spectrum antibiotics were reduced remarkably after the intervention. The quality of drug treatment was improved and patient drug expenses was continuously reduced.

**Conclusion:** Giving full play to the functions of the DTC can significantly improve the level of drug treatment and reduce unreasonable drug use to save unnecessary drug expenses and slow the development of drug resistance.

## Introduction

The Drug and Treatment Committee (hereinafter referred to as the DTC) is at the top of the hierarchy of hospital pharmacy management (Tyler et al., 2008). Previously, its members were primarily pharmaceutical personnel. The Committee’s main function was to guide the supply and allocation of drugs in the hospital according to the overall development plan and policies of the hospital. This function took the form of organizing regular meetings and discussions. This arrangement led to the failure of the pharmaceutical commission to play the overall decision-making function of the drug management center, especially the role of promoting rational drug use and rational use of antibiotics (Webb, 2012). The irrational use of antibiotics in China is more prominent than the rational use. First, irrational use of antibiotics increases the incidence of adverse drug reactions and drug-induced diseases. Second, it leads to increased bacterial drug resistance, resulting in the continuous reduction in effectiveness or even failure of some typically effective antibiotics. This combination not only affects the treatment of the disease, but also increases the economic burden of patients and objectively contributes to the unreasonable rise of medical expenses.

China established the national DTC in March 2022 to further strengthen the pharmaceutical administration of medical institutions, promote rational drug use, and give full play to the role of expert technical support. The main responsibilities of the Committee include: studying the development status of pharmaceutical management in medical institutions and proposing policy suggestions; providing technical support for the establishment and improvement of drug selection, procurement, use, and evaluation systems in medical institutions; promoting the implementation of clinical diagnosis and treatment guidelines related to drug treatment and guiding principles for clinical application of drugs; promoting the establishment and improvement of China’s pharmaceutical care system; strengthening the instruction of pharmacists and standardizing pharmaceutical care; and investigating and manage major mass drug accidents.

Beginning in 2011, our hospital optimized the organizational structure, division of labor, management functions, and work priorities of its DTC. The main purpose of the DTC is to promote the formulation and implementation of clinical diagnosis and treatment guidelines related to drug treatment and guiding principles for clinical application of drugs, monitoring, and evaluating the use of drugs in the institution, proposing intervention and improvement measures, and guiding clinical rational drug use (Lloyd et al., 2021; Zaragoza Laura Largeau et al.). Before 2016, the DTC had no sub groups. The DTC is composed of the heads of medical, pharmaceutical, infectious diseases, clinical microbiology, nursing, hospital infection management, and other departments and personnel with relevant professional senior technical post qualifications. Medical, pharmaceutical, and other departments are jointly responsible for daily management of the duties of the DTC. The antimicrobial stewardship group was established under the DTC in 2016.

## Methods

### Design and setting

This study was conducted in China at the Shandong Provincial Third Hospital, Shandong University, a 1,400-bed tertiary university teaching hospital. The members of the DTC are personnel from the medical, pharmaceutical, and nursing departments and clinical medical experts. The specific work is organized and implemented by the medical and pharmaceutical departments. The primary management function of the DTC in our hospital was the control of irrational drug use and antimicrobial stewardship beginning in 2016. Typical management cases, bacterial resistance rates, irrational drug use, and drug expenditures from 2016 to 2021 were summarized. We separated the study duration into two periods according to the time of introduction of antimicrobial stewardship (beginning January 2016): Before intervention: 1 year before the introduction (from January 2015 to December 2015); after intervention: 6 years after the introduction (from January 2016 to December 2021).

### Aim of the study

This study aimed to investigate the key points in the transformation of the functions of the DTC and how to provide full play to its role in the control of rational drug use, especially in the management of antibiotic use.

### Organization and responsibilities of the drug and therapeutics committee

**FIGURE 1:**
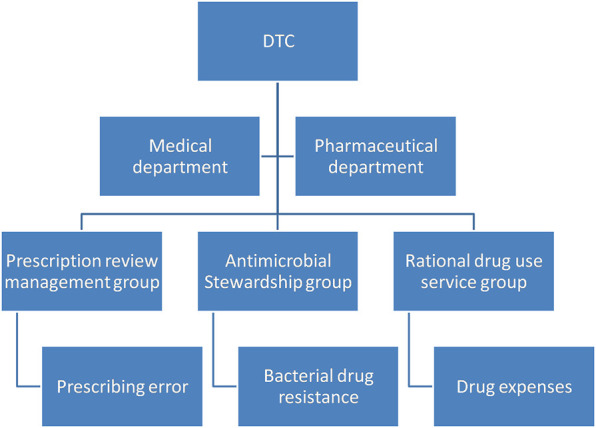
Composition of the management team of the DTC.

To better promote rational drug use, six working groups were established under the DTC. The main responsibilities included the following: selection of hospital drug variety, analysis and evaluation of drug risks, monitoring and analysis of drug use, and evaluation of rational drug use and antimicrobial stewardship. Among these groups, the prescription review management group, antimicrobial stewardship group, and rational drug use service group were responsible for monitoring rational drug use and antimicrobial stewardship (Figure 1).

The main tasks of the antimicrobial stewardship group were to review all antibiotic prescriptions for information related to, for example, indications, time of dose, and dosing density, duration, and route. Real-time recommendations were provided by the antimicrobial stewardship team for correcting antibiotic choice, density, duration, and route based on microbiological results and treatment protocols. Monthly multidisciplinary antibiotic rounds were undertaken in all departments. Antimicrobial stewardship team members reported inappropriate antibiotic use to hospital and department leaders monthly. Real-time information on antibiotic resistance was reported on the hospital information system.

The prescription review team evaluated prescriptions every month for the rational use of drugs and comments on cases of unreasonable use were directed to the physician. The prescription review team paid attention to the delivery of understandable information, established a preliminary prescription audit system to monitor drug use, and intervened in a timely manner in cases of unreasonable drug use.

The rational drug use service group organized clinical pharmacy-related training to improve the level of rational drug use. The DTC organized Committee members and clinical medicine and clinical pharmacy experts to conduct dynamic monitoring of prescribed medications, regularly sampling prescriptions or cases for reasonable evaluation. The evaluation results have been reported (Yang et al., 2021; Pallares et al., 2022). The types of irrational drug use, DTC interventions, and outcomes of the intervention were recorded by clinical pharmacists. In addition, the effect of rational use of antibiotics was evaluated with changes in trends of bacterial drug resistance as measured with the minimal inhibitory concentration method.

### Data analysis

Statistics were performed on the rate of rational drug use and the rate of antibiotic utilization, with a focus on monitoring the proportion of drug use and drug costs to evaluate the effect of the intervention. The regression equation was obtained with SPSS Linear-by-Linear Association and linear regression. Trend analysis was performed and Student’s t tests were calculated using SPSS version 22. The accepted significance level for all hypothesis contrasts was 0.05.

## Results

### Irrational drug use

Instances of irrational drug use and their causes and proposed interventions to counter them were categorized according to a simplified form of the Pharmaceutical Care Network Europe drug-related problem classification (PCNE-DRP), version 9.0. Serious prescribing errors that required correction related to inappropriate prescription, missing drug indications, inappropriate drug combinations, combinations with herbal medications or dietary supplements, over-prescription of drugs, and errors related to the dose, frequency, and duration of treatment. The types of irrational drug use, DTC interventions, and outcomes of the intervention are detailed in Table 1.

**TABLE 1:** Types of irrational drug uses and DTC interventions.

The cause	Frequency (%)						
	2015	2016	2017	2018	2019	2020	2021
C1 Drug selection							
C1.1 Inappropriate drug according to guidelines/formulary	39 (13.59)	32 (13.28)	29 (14.36)	27 (15.34)	24 (16.44)	18 (15.93)	16 (19.51)
C1.2 Inappropriate drug (within guidelines but otherwise contra-indicated)	22 (7.67)	17 (7.05)	15 (7.43)	11 (6.25)	9 (6.16)	6 (5.31)	2 (2.44)
C1.3 No indication for drug	23 (8.01)	17 (7.05)	15 (7.43)	12 (6.82)	11 (7.53)	10 (8.85)	6 (7.32)
C1.4 Inappropriate combination of drugs, drugs and herbal medications, or drugs and dietary supplements	31 (10.8)	29 (12.03)	24 (11.88)	21 (11.93)	17 (11.64)	13 (11.5)	9 (10.98)
C1.7 Too many drugs prescribed for indication	33 (11.5)	31 (12.86)	27 (13.37)	21 (11.93)	16 (10.96)	12 (10.62)	8 (9.76)
C3 Dose selection							
C3.1 Drug dose too low	9 (3.14)	7 (2.90)	8 (3.96)	6 (3.41)	5 (3.42)	5 (4.42)	4 (4.88)
C3.2 Drug dose too high	22 (7.67)	18 (7.47)	16 (7.92)	15 (8.52)	11 (7.53)	9 (7.96)	7 (8.54)
C3.3 Dosage regimen not frequent enough	12 (4.18)	7 (2.9)	6 (2.97)	6 (3.41)	4 (2.74)	2 (1.77)	2 (2.44)
C3.4 Dosage regimen too frequent	33 (11.50)	28 (11.62)	21 (10.40)	17 (9.66)	14 (9.59)	9 (7.96)	8 (9.76)
C4 Treatment duration							
C4.1 Duration of treatment too short	22 (7.67)	16 (6.64)	9 (4.46)	8 (4.55)	6 (4.11)	6 (5.31)	6 (7.32)
C4.2 Duration of treatment too long	23 (8.01)	22 (9.13)	18 (8.91)	17 (9.66)	15 (10.27)	11 (9.73)	7 (8.54)
C9 Other							
C9.1 No or inappropriate outcome monitoring (incl. Therapeutic drug monitoring)	18 (6.27)	17 (7.05)	14 (6.93)	15 (8.52)	14 (9.59)	12 (10.62)	7 (8.54)
Total	287	241	202	176	146	113	82
DTC Interventions							
I1 At prescriber level							
I1.1 Prescriber informed only	82 (28.57)	50 (20.75)	33 (16.34)	29 (16.48)	24 (16.44)	17 (15.04)	15 (18.29)
I1.2 Prescriber asked for information	55 (19.16)	34 (14.11)	25 (12.38)	20 (11.36)	15 (10.27)	14 (12.39)	12 (14.63)
I1.3 Intervention proposed to prescriber	78 (27.18)	99 (41.08)	88 (43.56)	84 (47.73)	72 (49.32)	64 (56.64)	41 (50)
I1.4 Intervention discussed with prescriber	72 (25.09)	58 (24.07)	56 (27.72)	43 (24.43)	35 (23.97)	18 (15.93)	14 (17.07)
I3 At drug level							
I3.1 Drug changed to …	86 (29.97)	79 (32.78)	52 (25.74)	45 (25.57)	37 (25.34)	32 (28.32)	27 (32.93)
I3.2 Dosage changed to …	52 (18.12)	48 (19.92)	47 (23.27)	42 (23.86)	34 (23.29)	24 (21.24)	17 (20.73)
I3.3 Formulation changed to …	22 (7.67)	19 (7.88)	29 (14.36)	23 (13.07)	22 (15.07)	18 (15.93)	12 (14.63)
I3.4 Instructions for use changed to …	34 (11.85)	27 (11.2)	16 (7.92)	12 (6.82)	8 (5.48)	8 (7.08)	6 (7.32)
I3.5 Drug paused or stopped	49 (17.07)	38 (15.77)	30 (14.85)	28 (15.91)	21 (14.38)	17 (15.04)	10 (12.2)
I3.6 Drug started	44 (15.33)	30 (12.45)	28 (13.86)	26 (14.77)	24 (16.44)	14 (12.39)	10 (12.2)
Intervention Acceptance							
A1 Intervention accepted	227 (79.09)	197 (81.74)	171 (84.65)	151 (85.8)	131 (89.73)	105 (92.92)	78 (95.12)
A1.1 Intervention accepted and fully implemented	121 (42.16)	113 (46.89)	110 (54.46)	115 (65.34)	119 (81.51)	92 (81.42)	72 (87.8)
A1.2 Intervention accepted, partially implemented	49 (17.07)	52 (21.58)	40 (19.80)	23 (13.07)	8 (5.48)	8 (7.08)	2 (2.44)
A1.3 Intervention accepted but not implemented	57 (19.86)	32 (13.28)	21 (10.4)	13 (7.39)	4 (2.74)	5 (4.42)	4 (4.88)
A2 Intervention not accepted	60 (20.91)	44 (18.26)	31 (15.35)	25 (14.2)	15 (10.27)	8 (7.08)	4 (4.88)
A2.1 Intervention not accepted: not feasible	31 (10.8)	26 (10.79)	22 (10.89)	17 (9.66)	10 (6.85)	6 (5.31)	3 (3.66)
A2.2 Intervention not accepted: no agreement	29 (10.10)	18 (7.47)	9 (4.46)	8 (4.55)	5 (3.42)	2 (1.77)	1 (1.22)
Status of the drug-related problem							
O1 Problem solved	138 (48.08)	133 (55.19)	125 (61.88)	118 (68.1)	115 (78.77)	90 (79.65)	66 (80.49)
O2 Problem partially solved	32 (11.15)	32 (13.28)	25 (12.38)	20 (10.43)	12 (8.22)	10 (8.85)	8 (9.76)
O3 Problem not solved	117 (40.77)	76 (31.54)	52 (25.74)	38 (21.47)	19 (13.01)	13 (11.50)	8 (9.76)

C: causes, I: interventions, A: acceptance, O: Status of the drug-related problem.

The DTC intervention led to a significant reduction in prescribing errors (Table 1). The prescribing errors decreased by 71.43% from 2015 (287 cases) to 2021 (82 cases) (*p* < 0.05). The regression equation of prescribing error was: y = 33.107x + 310.57 (F = 1958.07, *p* < 0.05; t = −44.25, *p* < 0.05; *R* ^2^ = 0.9934), indicating a linear downward trend. The intervention acceptance rate increased by 16.03% from 2015 (79.09%) to 2021 (95.12%) (*p* < 0.05). The regression equation of the intervention acceptance rate was: y = 2.6975x + 76.217 (F = 285.31, *p* < 0.05; t = 16.89, *p* < 0.05; *R* ^2^ = 0.9908), indicating a linear upward trend. The problem solved rate increased by 32.41% from 2015 (48.08%) to 2021 (80.49%) (*p* < 0.05). The regression equation of the problem solved rate was: y = 5.823x + 44.16 (F = 43.32, *p* < 0.05; t = 6.58, *p* < 0.05; *R* ^2^ = 0.9416), indicating a linear upward trend. Irrational drug use continuously reduced; the level of drug treatment improved.

### Continuous optimization of rational drug use indicators

**FIGURE 2:**
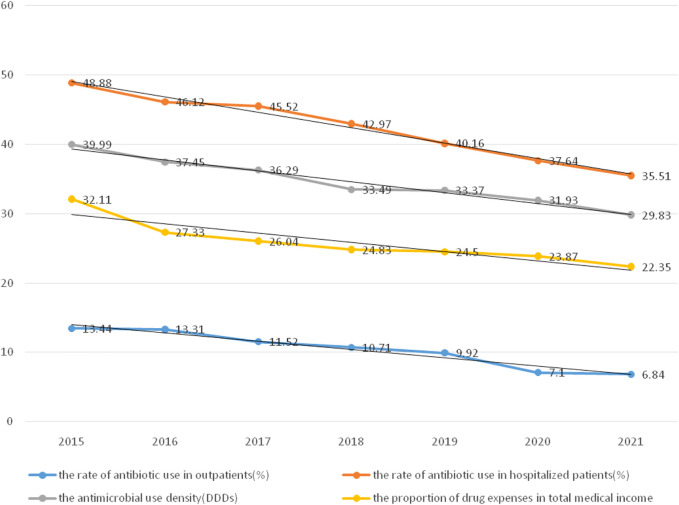
The utilization rate of antibiotics and the proportion of drug expenses from 2015 to 2021.

Goals related to the previous ones were to continuously strengthen the management of rational drug use through timely intervention by the DTC, manage clinical cases of excessive use of antibiotics or adjuvant drugs, and strengthen the optimal control of indicators. From 2016 to 2021, the scale of the hospital continued to expand (from 800 to 1,400 beds) and the number of patients with severe diseases and disorders continued to increase. However, the rate of antibiotic utilization was stably controlled, as was the proportion of drug expenses related to antibiotics (Figure 2).

The rate of antibiotic use in hospitalized patients decreased by 13.37% from 2015 (48.88%) to 2021 (35.51%) (*p* < 0.05). The rate of antibiotic use in outpatients decreased by 6.6% from 2015 (13.34%) to 2021 (6.84%) (*p* < 0.05). The antimicrobial use density decreased by 10.16% from 2015 (39.99%) to 2021 (29.83%) (*p* < 0.05). The proportion of drug expenses in the total medical expenditures decreased by 9.76% from 2015 (32.11%) to 2021 (22.35%) (*p* < 0.05).

### Antibiotics resistance rate

**FIGURE 3:**
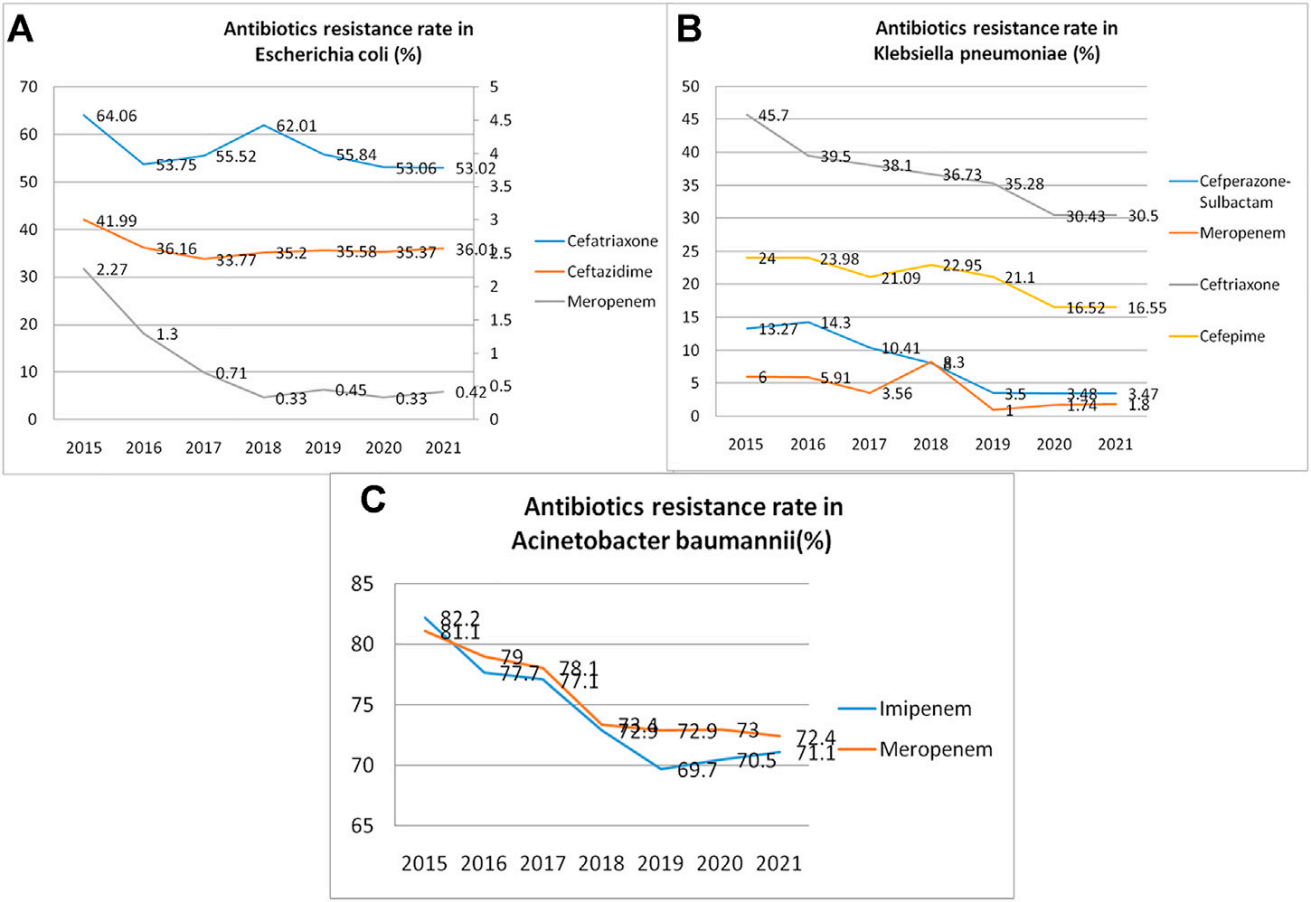
Antibiotic resistance rates (per year) reduced remarkably during the study period. **(A)**. Resistance rate of three antibiotics commonly used in *Escherichia coli* infection*;* **(B)**. Resistance rate of four antibiotics commonly used in *Klebsiella pneumonia* infection*;* **(C)**. Resistance rates of two antibiotics commonly used in *Acinetobacter baumannii* infection.

The resistance rate of general spectrum antibiotics reduced remarkably after intervention. Resistance rates of three commonly used antibiotics (cefatriaxone, ceftazidime, and meropenem) in *Escherichia coli* were significantly lower after intervention than those before intervention (64.06 vs. 53.02%, 41.99 vs. 36.01%, 2.27 vs. 0.42%; all *p* < 0.05) (Figure 3A). Resistance rates of commonly used antibiotics (cefepime, ceftriaxone, meropenem, and cefoperazone-sulbactam) in *Klebsiella pneumoniae* were significantly lower after intervention than those before intervention (24.00 vs. 16.55%, 45.7 vs. 30.5%, 6.00 vs. 1.8%, 13.27 vs. 3.47%; all *p* < 0.05) (Figure 3B). Resistance rates of imipenem and meropenem in *Acinetobacter baumannii* were reduced by the intervention (82.2 vs. 71.1%, 81.1 vs. 72.4%; all *p* < 0.01) (Figure 3C) (Figure 3).

## Discussion

The DTC of our hospital was established in 2002 with a primary function of ensuring drug supply. However, with the transformation of pharmaceutical functions in Chinese hospitals, the focus of the hospital pharmacy should change from ensuring drug supply to strengthening rational drug use and pharmaceutical technical services. In contrast, the phenomenon of irrational drug use in hospitals and the excessive use of antibiotics are becoming more and more obvious. An effective institution is needed to control irrational drug use. Therefore, the management function of the DTC in our hospital changed beginning in 2016, focusing on monitoring the rationality of clinical medication use, especially antimicrobial stewardship (Ribeiro-Vaz et al., 2016;and Brooks, 2010).

In recent years, medical institutions have actively implemented China’s policy on the rational clinical application of antibiotics and strengthened the control of nosocomial infections. Under the guidance of the DTC, with the standardized management and rational application of antibiotics in hospital, the strengthening of communication ability between laboratory and clinic, and the strengthening of awareness of prevention and control of drug-resistant bacterial infection, the spread of drug-resistant bacteria has been curbed to a certain extent (Zhang et al., 2018; Yang et al., 2020). The goals of rational drug use and antimicrobial stewardship are to reduce improper drug use, improve drug treatment level, reduce drug expenditures, and delay bacterial drug resistance, so as to better protect the safety of patients. As the organization responsible for the management of rational drug use and antimicrobial stewardship, the DTC needs a stable management department to coordinate relevant work. The pharmacy department is not presently competent for relevant responsibilities. Thus, the cooperation of medical and other departments is needed to determine the division of labor and truly ensure the smooth development of rational drug use hospital-wide (Björkman et al., 2007; Ciccarello et al., 2021). The joint efforts and full cooperation of all members of the DTC are the basis for effective rational drug use (Alefan et al., 2019).

In terms of specific work measures, the DTC should promote the improvement of drug treatment planning through functional management and expert cooperation. In addition, the DTC should ensure the rational use of drugs through index monitoring and rational drug use evaluation (Plet et al., 2013; Religioni and Pakulska, 2020). Therefore, we should have various comprehensive management measures in addition to departmental cooperation to better realize this drug management function (Gustafsson et al., 2011; Matlala et al., 2015). Moreover, the DTC should also be supported by a convenient pharmaceutical management statistics information platform (Durán-García et al., 2011).

One role of the DTC should be to urge doctors to use drugs rationally. This is needed because although most doctors abide by the norms, there are always a few who do not fully regulate the use of drugs for various reasons. In the early stages of the DTC, our hospital continuously strengthened management and achieved good results. However, the next step should start with improving the organizational form of the pharmaceutical Committee and strengthening its monitoring, analysis, decision-making, accountability, and other responsibilities so that the use of drugs is reasonably monitored. Likewise, we will continue to track and rectify any abnormal conditions found, clarify the responsibilities of the department in the management of doctors, help them find clues to illegal drug use, help them carry out management, and continuously promote the Plan-Do-Check-Act cycle. With the continuous development of the expert autonomous pharmaceutical Committee, the spirit of self-discipline in doctors will continue to strengthen. Ultimately, drugs will be effective in the continuous improvement of medical technology.

## Conclusions

Intervention by the DTC, as the guardian of safe and rational drug use, led to a significant reduction in prescribing errors. The DTC established a monitoring and long-term management mechanism for the rational use of antibiotics, improved society’s understanding of the harm of antibiotic abuse, and worked hard to maintain the health of the population. In conclusion, the implementation of the DTC in our hospital reduced medical expenses, improper use and abuse of drugs, and antibiotic resistance rates. However, further efforts are needed to improve the use of antibiotics. Based on our experience, it is strongly recommended to implement a DTC in all local hospitals in China.

## Data Availability

The data that support the findings of this study are available from the corresponding author upon reasonable request.
